# The comparable efficacy of denosumab on bone mineral density in rheumatoid arthritis patients with postmenopausal osteoporosis: A retrospective case-control study

**DOI:** 10.1097/MD.0000000000034219

**Published:** 2023-06-30

**Authors:** Seong-Kyu Kim, Ji-Won Kim, Hwajeong Lee, Sung-Hoon Park, Jung-Yoon Choe, Boyoung Kim

**Affiliations:** a Division of Rheumatology, Department of Internal Medicine, Catholic University of Daegu School of Medicine, Daegu, Republic of Korea.

**Keywords:** bisphosphonate, BMD, denosumab, osteoporosis, SERMs

## Abstract

Little is known about differences in the therapeutic efficacy of denosumab in subjects with and without rheumatoid arthritis (RA). This study compares the changes in bone mineral density (BMD) between RA patients and controls without RA who had been treated with denosumab for 2 years for postmenopausal osteoporosis. A total of 82 RA patients and 64 controls were enrolled, who were refractory to selective estrogen receptor modulators (SERMs) or bisphosphonates and completed the treatment of denosumab 60 mg for 2 years. The efficacy of denosumab in RA patients and controls was assessed using areal BMD (aBMD) and T-score of the lumbar spine, femur neck, and total hip. A general linear model with repeated measures analysis of variance was used to determine differences in aBMD and T-score between 2 study groups. No significant differences in percent changes in aBMD and T-scores by denosumab treatment for 2 years at the lumbar spine, femur neck, and total hip were evident between RA patients and controls (*P* > .05 of all), except T-score of the total hip (*P* = .034). Denosumab treatment equally increased aBMD at the lumbar spine and T-scores at the lumbar spine and total hip between RA patients and controls without statistical differences, but RA patients showed less improvement in aBMD at the femur neck (*p*_time*group_ = 0.032) and T-scores at the femur neck and total hip than controls (*p*_time*group_ = 0.004 of both). Changes in aBMD and T-scores after denosumab treatment in RA patients were not affected by previous use of bisphosphonates or SERMs. Differences of T-score at the femur neck among previous bisphosphonate users and aBMD and T-score at the femur neck and T-scores at the total hip were evident. This study revealed that 2 years of denosumab treatment in female RA patients achieved comparable efficacy on BMD to controls at the lumbar spine, but showed somewhat insufficient improvement at the femur neck and total hip.

## 1. Introduction

Rheumatoid arthritis (RA) is a chronic autoimmune disease that affects approximately 1% of the general population and is characterized by articular inflammation and involvement of extra-articular organs, subsequently leading to substantial joint pain, functional disability, and increased mortality and morbidities.^[1,2]^ The prevalence of RA is 2 to 3 times higher in women than in men. In addition to intra-articular manifestations of synovial hyperplasia, bone erosion, and cartilage destruction, alternation of the microarchitecture and macro-architecture of the bone such as lower bone mass, decline of bone mineral density (BMD), osteoporosis, and fractures in RA have been well recognized.^[3–5]^ Advanced understanding of the underlying pathogenesis of RA has contributed to the development of diverse therapeutic agents such as cytokine inhibitors, B cell inhibitor, co-stimulation blocker, or Janus kinase inhibitors to reduce inflammation, structural damage of the joints, and irreversible disability.^[6]^

Osteoporosis is a metabolic bone disorder characterized by low BMD and impaired microarchitectural bone structure due to disruption of the balance between bone formation and resorption, resulting in increased bone fragility and fracture risk.^[7]^ Accumulating understanding of diverse skeletal signal pathways including Wnt signaling for bone formation and the receptor activator of nuclear factor-κB ligand (RANKL)-RANK system for bone resorption has provided clues for the development of therapeutic agents against osteoporosis.^[8,9]^ Currently available and effective anti-osteoporotic drugs including bisphosphonates, selective estrogen receptor modulators (SERMs), and teriparatide potentially lead to decrease the risk of bone fracture.^[10,11]^ Recently, monoclonal antibody treatments that block specific targets such as RANKL and sclerostin involved in the pathogenesis of osteoporosis have been used in clinical practice. Especially, the RANK-RANKL system is crucially responsible for the activation of osteoclasts involved in bone resorption in the pathogenesis of osteoporosis.^[9]^ Denosumab is a fully humanized monoclonal antibody that inhibits bone resorption through blockage of osteoclast activity by competitive binding to RANKL.^[12]^ The FREEDOM trial demonstrated that treatment with 60 mg of denosumab significantly decreased the risk of vertebral, non-vertebral, and hip fractures in postmenopausal women with osteoporosis, compared with controls.^[13]^ Furthermore, the FREEDOM trial and an open-label extension study found that the therapeutic effect of denosumab continued even after 10 years of follow-up.^[14]^

The prevalence of osteoporosis in RA patients is estimated to be approximately twice that of the general population.^[5,15]^ A meta-analysis revealed that the prevalence of osteoporosis among those with RA was estimated at approximately 27.6%.^[16]^ It suggests that chronic inflammatory diseases such as RA might be considered an independent risk factor for osteoporosis. Recent data from 2 meta-analyses of the efficacy of denosumab in RA patients with osteoporosis revealed that denosumab treatment led to increased BMD of the lumbar spine and total hip compared to controls treated with placebo or bisphosphonates.^[17,18]^ Our hypothesis is that the efficacy of denosumab treatment may be reduced in RA patients with chronic inflammation compared to controls without RA. However, no data comparing the difference in treatment efficacy of denosumab between RA patients and controls without RA are available. Here, we evaluated whether 2 years of denosumab treatment improved BMD in RA patients with postmenopausal osteoporosis compared to controls without RA.

## 2. Subjects and methods

### 2.1. Study population

This study retrospectively enrolled female RA patients who met the RA classification criteria proposed by the American College of Rheumatology/European League Against Rheumatism in 2010^[19]^ and also met the diagnostic criteria for postmenopausal osteoporosis with lumbar spine or total hip BMD T-score of <−2.5 proposed by World Health Organization and modified by the International Osteoporosis Foundation by dual-energy X-ray absorptiometry (DXA).^[20,21]^ In addition, gender- and age-matched female subjects with postmenopausal osteoporosis were recruited as controls. Controls were excluded if they had a medical history of diagnosis or treatment of other inflammatory or autoimmune diseases such as RA, systemic lupus erythematosus, Sjogren syndrome, pseudogout, or psoriatic arthritis.

**Figure 1.**
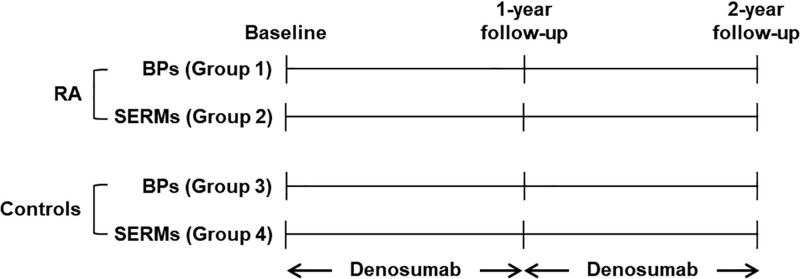
Scheme for each group of study population. BPs = bisphosphonates, RA = rheumatoid arthritis, SERMs = selective estrogen receptor modulators.

Both RA patients and controls used either oral bisphosphonates or SERMs as antiresorptive agents against postmenopausal osteoporosis for at least 1 year and then switched to denosumab following lack of treatment efficacy due to BMD not reaching a T-score > −2.5.^[22]^ All participants initiated denosumab treatment between April 2018 and October 2020 and then received subcutaneous injections of 60 mg of denosumab every 6 months for at least 2 years. Subjects who had been previously exposed to denosumab treatment were excluded. As shown Figure 1, the study groups were divided into RA patients and controls. Each group was further subdivided into 2 groups according to use of anti-osteoporotic oral bisphosphonates (group 1 in RA and group 3 in controls) and SERMs (group 2 in RA and group 4 in controls) immediately before the use of denosumab.

This study was approved by the Institutional Review Board at Daegu Catholic University Medical Center (IRB No. CR-23-029). Informed consent of RA patients and controls in this study was waived because this study had a retrospective design.

### 2.2. Clinical and laboratory information

Clinical data of all study participants including age (years), disease duration (years), previous fracture history, body mass index (BMI, kg/m^2^), smoking status, and alcohol consumption were collected. Fracture history was defined as previous or current fracture of the lumbar spine or femur associated with osteoporosis. Smoking and alcohol consumption status was based on whether a participant was currently smoking or drinking. At the time of study, the presence of comorbidities such as hypertension, diabetes mellitus, and thyroid diseases and disease duration of osteoporosis were investigated through a review of medical records. Laboratory markers, including erythrocyte sedimentation rate (ESR), C-reactive protein (CRP), calcium, and vitamin D, were also assessed at the time of denosumab initiation. Anti-osteoporotic medications including alendronate, ibandronate, and risedronate for bisphosphonates and raloxifene and bazedoxifene for SERMs used immediately before denosumab initiation were identified for statistical analysis. The use of corticosteroid and dosage of corticosteroid (mg/day) was identified. In addition, the use of disease-modifying anti-rheumatic drugs (DMARDs) at baseline was identified. Conventional DMARDs included methotrexate, hydroxychloroquine, sulfasalazine, or leflunomide. The use of biologic DMARDs including abatacept, tocilizumab, adalimumab, and etanercept was also identified. We measured composite disease activity indices for RA including Disease Activity Score 28 joints using ESR (DAS28-ESR) and using CRP (DAS28-CRP).

### 2.3. Measurement of BMD

Areal BMD (aBMD, g/cm^2^) measurement was performed at the lumbar spine, femur neck, and total hip using DXA (Horizon DXA system, Hologic Inc., Marlborough, MA) at the Department of Radiology, Daegu Catholic University Medical Center. All results of aBMD assessments measured at 3 time points (at baseline [i.e., at the time of denosumab initiation], 1-year follow-up after denosumab treatment, and 2-year follow-up after denosumab treatment) were identified. T-scores for the lumbar spine, femur neck, and total hip were assessed by the National Health and Nutrition Examination Survey III reference database using young Caucasian females as the reference population.^[23,24]^

### 2.4. Outcomes

Primary outcome of this study was percent changes (%) in aBMD of the lumbar spine, femur neck, and total hip from baseline to the 1-year follow-up and 2-year follow-up assessments in RA patients and controls. Secondary outcomes were differences in aBMD and T-scores between RA and controls after 2 years of denosumab treatment, differences in aBMD and T-scores between RA patients using bisphosphonates or SERMs prior to denosumab treatment (group 1 vs group 2), and differences in aBMD and T-scores between RA patients and controls according to either use of bisphosphonates (group 1 vs group 3) or SERMs (group 2 vs group 4) prior to denosumab treatment.

### 2.5. Statistical analysis

Descriptive data were described as the median (interquartile range) for continuous parameters and percentage (%) for categorical parameters, respectively. The normality of data distribution was assessed using Kolmogorov–Smirnov test, with a non-parametric test performed for further analysis. Sample size calculation was performed with the power calculator from the G Power program (http://www.gpower.hhu.de) with the 2 independent groups with α set at 0.05 and power at 0.85.

Differences in baseline parameters of age, gender, BMI, ESR, CPR, calcium, vitamin D, aBMD, and T-scores were assessed with Mann–Whitney *U* tests. Differences in previous fractures, smoking, alcohol consumption, comorbidities, and anti-osteoporotic drugs were assessed with chi-square tests. A general linear model with repeated measures analysis of variance was used to determine differences in aBMD and T-scores between 2 groups, considering interactions of follow-up duration and disease groups (RA vs controls) or baseline treatment groups (SERMs vs bisphosphonates). A *P* value < .05 was considered statistically significant. Statistical analysis was performed using SPSS version 19.0 (SPSS Inc., Chicago, IL). The plots in the figures presented in this study were generated by GraphPad Prism 5.0 (GraphPad Software, Inc., San Diego, CA).

## 3. Results

### 3.1. Baseline characteristics of study population

Baseline characteristics of subjects participating in this study were described in Table 1. All RA patients (n = 82) and controls (n = 64) who enrolled in this study were female. There were no significant differences in age, smoking, alcohol consumption, BMI, comorbidities, or disease duration of osteoporosis between 2 groups (*P* > .05 for all), except for previous fractures, which were more frequent in RA patients than in controls (*P* = .014). No significant difference in the use of SERMs or bisphosphonates immediately before denosumab administration was noted between 2 groups (*P* = .135). In terms of laboratory markers, ESR, CRP, and calcium levels were similar between 2 groups (*P* > .05 for all), but vitamin D level was higher in controls than in RA patients (*P* < .001). No differences in aBMD and T-scores of the lumbar spine, femur, and total hip were noted between RA patients and controls at the time of study enrollment. Median values of disease activity indices including DAS28-ESR and DAS28-CRP were 2.34 and 1.77, respectively. In addition, anti-rheumatic medications including conventional DMARDs, biologic DMARDs, and corticosteroid received at enrollment were identified in RA patients. Nine patients with RA used the biologic DMARDs such as abatacept (n = 3), tocilizumab (n = 3), adalimumab (n = 2), or etanercept (n = 1).

**Table 1:** Baseline characteristics of study population.

Parameters	RA (n = 82)	Control (n = 64)	*P* value
Age (yr)	72.0 (66.8, 80.0)	74.5 (67.0, 81.8)	.314
Disease duration (yr)	15.8 (8.6, 23.4)		
Previous fracture, n (%)	10 (12.2)	1 (1.6)	.014
Body mass index (kg/m^2^)	22.2 (20.0, 24.4)	22.7 (20.4, 25.5)	.103
Smoking, n (%)	0 (0.0)	2 (3.1)	.190
Alcohol consumption, n (%)	2 (2.4)	3 (4.7)	.384
Comorbidities			
Hypertension, n (%)	41 (50.0)	30 (46.9)	.418
Diabetes mellitus, n (%)	7 (15.6)	7 (8.5)	.144
Thyroid diseases, n (%)	7 (8.5)	10 (15.6)	.144
Disease duration of osteoporosis (yr)	10.5 (6.71, 13.4)	10.2 (6.71, 15.39)	.782
Previous anti-osteoporotic drugs			.135
Previous SERMs, n (%)	30 (36.6)	16 (25.0)	
Previous bisphosphonates, n (%)	52 (63.4)	48 (75.0)	
Laboratory markers			
ESR (mm/h)	19.5 (9.8, 27.3)	16.0 (11.0, 25.0)	.497
CRP (mg/L)	1.10 (0.60, 2.80)	0.70 (0.60, 2.10)	.195
Calcium (mg/dL)	9.2 (9.0, 9.5)	9.2 (8.9, 9.4)	.351
Vitamin D (ng/mL)	22.4 (11.5, 31.8)	33.9 (25.7, 38.8)	<.001
Bone mineral density			
L-spine aBMD	0.70 (0.64, 0.75)	0.73 (0.66, 0.81)	.215
L-spine T-score	−2.95 (−3.23, −2.50)	−2.80 (−3.40, −2.00)	.122
Femur neck aBMD	0.51 (0.46, 0.56)	0.52 (0.48, 0.57)	.348
Femur neck T-score	−2.95 (−3.30, −2.50)	−2.80 (−3.40, −2.43)	.396
Total hip aBMD	0.63 (0.57, 0.68)	0.64 (0.59, 0.69)	.374
Total hip T-score	−2.30 (−2.80, −1.80)	−2.20 (−2.60, −1.63)	.302
Disease activity indices			
DAS28-ESR	2.34 (1.81, 3.57)		
DAS28-CRP	1.77 (1.29, 2.66)		
Corticosteroid, n (%)	72 (87.8)		
Dosage of corticosteroid (mg/d)	2.5 (1.3, 2.5)		
Medications, n (%)			
Methotrexate	62 (75.6)		
Hydroxychloroquine	25 (30.5)		
Sulfasalazine	23 (28.0)		
Leflunomide	5 (6.1)		
Biologics	9 (11.0)		

Values were described as median (interquartile range) or number (percentages).

Abbreviation: aBMD = areal bone mineral density, CRP = C-reactive protein, DAS28 = disease activity score 28 joints, ESR, erythrocyte sedimentation rate, RA = rheumatoid arthritis, SERMs = selective estrogen receptor modulators.

### 3.2. Comparison of changes in BMD after denosumab between RA patients and controls

We compared changes in BMD between RA patients and controls after denosumab initiation at the 1-year and 2-year follow-ups. Percent changes in aBMD and T-scores of the femur and total hip in controls indicated much higher efficacy than in RA patients after denosumab treatment at the 1-year follow-up (Table 2). However, no significant changes in aBMD and T-scores of the lumbar spine were observed between RA patients and controls at the 1-year follow-up, suggesting a similar efficacy of denosumab for the lumbar spine in even RA patients.

**Table 2:** Percent changes in bone mineral density after denosumab for 1 yr or 2 yr.

	1-yr follow-up			2-yr follow-up		
Bone mineral density	RA	Controls	*P* value	RA	Controls	*P* value
L-spine aBMD (%)	4.61 (1.27, 6.29)	3.28 (−0.69, 4.86)	.064	5.31 (1.35, 9.28)	4.92 (2.00, 9.41)	.903
L-spine T-score (%)	−5.56 (−13.78, 6.88)	−5.06 (−13.13, 8.47)	.954	−9.84 (−19.19, 3.75)	−8.96 (−22.96, −0.81)	.371
Femur neck aBMD (%)	1.15 (−3.39, 6.76)	4.45 (0.47, 11.13)	.017	4.13 (−1.43, 11.77)	6.03 (0.62, 13.46)	.448
Femur neck T-score (%)	0.00 (−8.00, 11.31)	−7.90 (−15.45, 3.26)	.003	−4.38 (−14.29, 8.87)	−9.55 (−15.94, 0.00)	.283
Total hip aBMD (%)	1.49 (−1.74, 6.06)	4.79 (0.19, 8.31)	.033	2.82 (−0.25, 7.04)	5.30 (0.93, 9.52)	.051
Total hip T-score (%)	0.00 (−8.52, 21.43)	−7.70 (−21.74, 4.50)	.002	−3.32 (−15.67, 20.26)	−10.26 (−22.48, 5.26)	.034

Values were described as median (interquartile range).

Abbreviations: aBMD = areal bone mineral density, RA = rheumatoid arthritis.

*P* values were calculated by Mann–Whitney *U* test.

With respect to changes in BMD at the 2-year follow-up, more percent changes in T-scores only at the total hip were measured in controls than in RA patients (−10.26% vs −3.32%, *P* = .034), indicating that denosumab treatment by controls had a more beneficial effect on BMD than in RA patients (Table 2). But denosumab treatment was not associated with a differential treatment efficacy between RA patients and controls in terms of aBMD and T-scores of the lumbar spine and femur neck and aBMD at the total hip.

**Figure 2.**
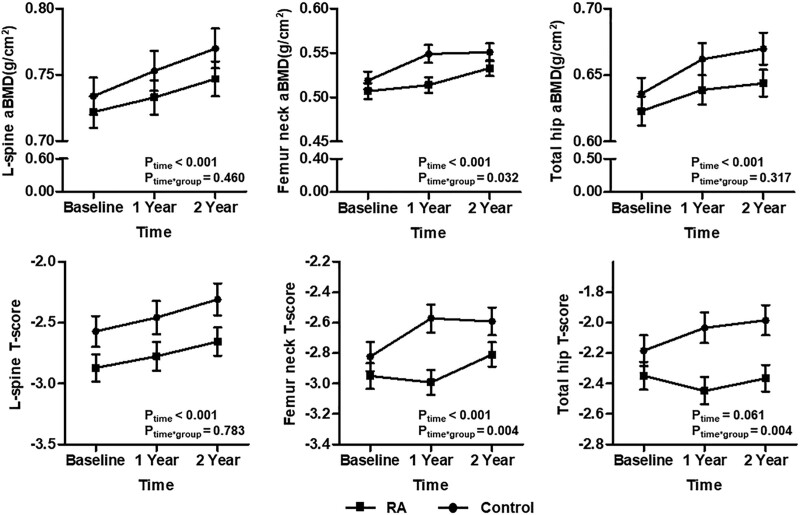
Comparison of changes in BMD between RA and controls treated with denosumab for 2 yr. Data in plots were presented as mean ± standard error. *P* values were assessed by a general linear model after consideration with time and study group. aBMD = areal bone mineral density, RA = rheumatoid arthritis.

Next, we assessed changes in BMD after denosumab treatment between 2 groups using a general linear model. Denosumab treatment in both RA patients and controls showed a gradual increase in aBMD of the lumbar spine, femur neck, and total hip (Fig. 2). Furthermore, no significant differences between RA patient and controls for aBMD of the lumbar spine and total hip were found (*p*_time*group_ = 0.460 and *p*_time*group_ = 0.317, respectively), whereas there was marked difference between 2 groups for aBMD of the femur neck (*p*_time*group_ = 0.032). However, significant improvement in T-scores of the femur neck and total hip in controls was noted compared with RA patients (*p*_time*group_ = 0.004 and *p*_time*group_ = 0.004, respectively), but not at the lumbar spine (*p*_time*group_ = 0.783).

### 3.3. Comparison of changes in BMD according to previous anti-osteoporotic medication use in RA patients

**Figure 3.**
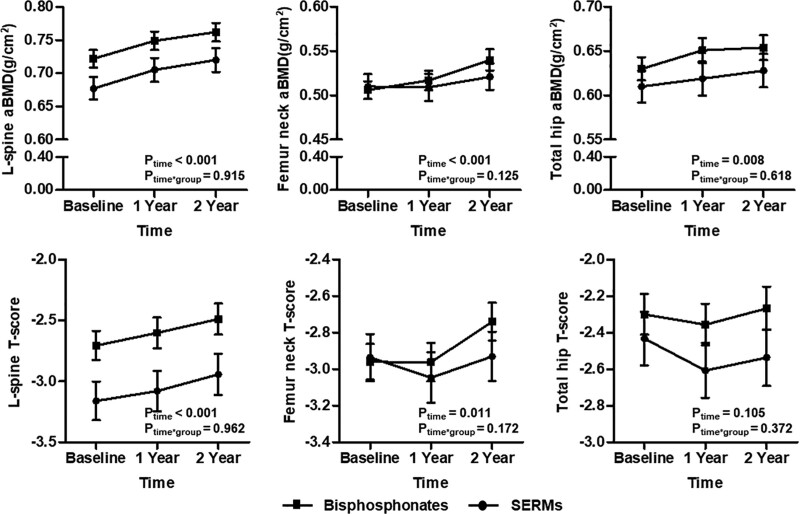
Comparison of changes in BMD between use of SERMs and bisphosphonates prior to denosumab for 2 yr in RA patients. Data in plots were presented as mean ± standard error. *P* values were assessed by a general linear model after consideration with time and study group. aBMD = areal bone mineral density, RA = rheumatoid arthritis, SERMs = selective estrogen receptor modulators.

We evaluated the differences in the therapeutic efficacy of denosumab treatment according to the use of bisphosphonates (group 1) or SERMs (group 2) before denosumab treatment in RA patients. As shown in Figure 3, anti-osteoporotic drugs used before denosumab treatment did not lead to a significant difference in aBMD and T-score improvement at the 3 measurement sites (*p*_time*group_ > 0.05 of all). Similarly, there were no difference in efficacy of denosumab according to the use of anti-osteoporotic drugs in controls (group 3 vs group 4) (data not shown).

### 3.4. Comparison of changes in BMD according to previous anti-osteoporotic drug use between RA patients and controls

**Figure 4.**
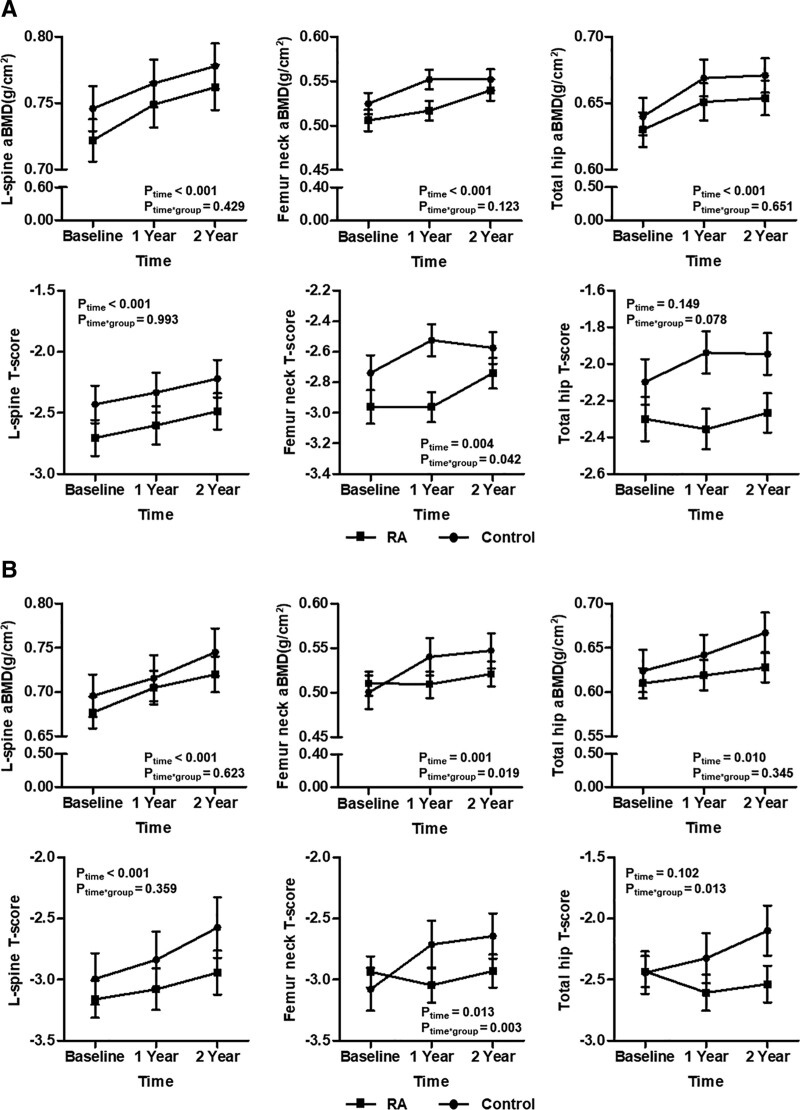
Comparison of changes in BMD between RA and controls according to use of bisphosphonates or SERMs prior to denosumab for 2 yr. (A) bisphosphonates and (B) SERMs Data in plots were presented as mean ± standard error. *P* values were assessed by a general linear model after consideration with time and study group. aBMD = areal bone mineral density, RA = rheumatoid arthritis, SERMs = selective estrogen receptor modulators.

We assessed the difference in the efficacy of denosumab treatment according to the use of bisphosphonates (group 1 vs group 3) or SERMs (group 2 vs group 4) before denosumab treatment between RA patients and controls. In a comparison of changes in BMD between RA patients and controls treated previously with bisphosphonates (group 1 vs group 3), only T-scores of the femur neck were significantly improved in controls treated with previous bisphosphonates compared with RA patients (*p*_time*group_ = 0.042), whereas T-scores of the lumbar spine and total hip did not show significant differences between 2 groups (Fig. 4A). In addition, controls showed no significant differences in aBMD improvement at the 3 sites compared with RA patients.

In patients previously treated with SERMs (group 2 vs group 4), controls showed much more improvement in aBMD of the femur neck, T-scores of that femur neck, and T-scores of the total hip than did RA patients (*p*_time*group_ = 0.019, *p*_time*group_ = 0.003, and *p*_time*group_ = 0.013, respectively) (Fig. 4B). But, there were no significant differences in aBMD of the lumbar spine and total hip and T-scores of the lumbar spine and between 2 groups.

## 4. Discussion

Denosumab, which exhibits strong antiresorptive activity, was approved for treatment of osteoporosis in 2010 and has demonstrated to be safe and effective in reducing the risk of vertebral, hip, and nonvertebral fractures, based on data from the FREEDOM trial and its extension study.^[13,14]^ A positive effect of denosumab has been confirmed for the treatment of postmenopausal osteoporosis as one of numerous comorbidities in patients with RA.^[17,18]^ However, whether the therapeutic efficacy of denosumab in RA patients is equivalent to that in a control group without inflammatory arthritis is uncertain. This study is the first to compare treatment efficacy between RA patients and controls based on changes in BMD using DXA and after denosumab treatment for 2 years. We found that denosumab treatment for 2 years switched from treatment with bisphosphonates or SERMs did not lead to significant differences in changes in aBMD and T-scores of the lumbar spine between RA patients and controls. Unlike BMD of the lumbar spine, a somewhat favorable effect of denosumab treatment on BMD of the femur neck or total hip in controls was evident compared with patients with RA.

Significant advances have been made in understanding the pathogenesis and treatment options of RA, and novel therapeutic agents targeting inflammatory molecules or signaling pathways related to the progression of RA have been developed.^[1,2,6]^ Bone erosion, together with synovial inflammation and cartilage degradation, is a pathologic process that presents in inflammatory synovial joints in RA. Evidence that osteoclast-like multinucleated giant cells are attached to eroded bone in inflammatory synovial joints suggests that osteoclasts play a crucial role in bone destruction in RA, which ultimately leads to joint deformity.^[25]^ RANK interaction with RANKL on osteoclast precursor cells promotes cellular differentiation into activated multinucleated osteoclasts and the survival of osteoclasts with the capacity for bone resorption.^[26,27]^ Osteoclasts that require activation of the RANK-RANKL signal pathway are also considered a potent therapeutic target in RA. Recent clinical studies have demonstrated that denosumab, a fully human monoclonal IgG2 antibody against RANKL, efficiently inhibits progression of bone erosion in RA as measured by significant reductions in modified total Sharp scores and modified Sharp erosion scores.^[17]^ A randomized, double-blind phase II trial found that MRI erosion scores and modified Sharp erosion scores at 6 months from baseline were lower in denosumab-treated group than in placebo-treated group in patients with RA.^[28]^ In the assessment of the efficacy of denosumab in 350 Japanese patients with RA, denosumab treatment markedly suppressed the progression of bone erosion at 12 months compared to placebo.^[29]^ In addition to the beneficial effect of denosumab on the progression of bone erosion, these studies also demonstrated that denosumab significantly increased mean percent changes in BMD of the lumbar spine, femur neck, or total hip compared with placebo. A recent meta-analysis of 10 studies of the efficacy of denosumab treatment involving a total of 1758 patients with RA found that denosumab treatment for 6 to 24 months led to an increase in BMD (5.12% at the lumbar spine, 2.72% at the total hip, and 2.20% at the femur neck).^[17]^ Another meta-analysis of 18 studies revealed improvements in change in BMD from baseline, such as 5.27% at the lumbar spine, 3.07% at the femur neck, and 2.82% at the total hip, after 12 months of denosumab treatment.^[18]^ Consistent with previous studies, we observed increased percent changes in aBMD of the lumbar spine, femur neck, and total hip after 1 year of denosumab treatment (4.61%, 1.15%, and 1.49%, respectively) and after 2 years (5.31%, 4.13%, and 2.82%, respectively). Based on these findings, denosumab treatment that inhibits RANNKL and sequentially blocks osteoclast activation, combined with other anti-rheumatic drugs, may help delay the loss of systemic bone mass and reduce the risk of fracture in patients with RA.

Proposed risk factors such as aging, estrogen deficiency, systemic inflammation, reduced mobility, glucocorticoid treatment, and sarcopenia are associated with the pathophysiology of osteoporosis or its related fractures.^[30]^ Inflammation has an adverse effect on BMD and it appears to be related to the activation of osteoclasts due to upregulation of osteoclastogenic cytokines, such as tumor necrosis factor-α, interleukin (IL)-6, and IL-17, during inflammatory processes. Several studies have suggested that treatment with biologic DMARDs is associated with a reduction in bone loss. Gulyás et al found that anti-TNF therapy for 12 months significantly reduced bone loss in the assessment using DXA with improvement of bone turnover markers and furthermore promoted bone formation.^[31]^ With respect to the effect of IL-6 inhibition on bone mass, bone destruction markers in patients treated with tocilizumab, an IL-6 receptor antagonist, combined with methotrexate were significantly decreased compared to those treated with methotrexate plus placebo.^[32]^ In addition to anti-rheumatic drugs, 2 recent meta-analyses show that denosumab treatment significantly induced an increase in BMD in patients with RA.^[17,18]^ Given that high inflammation is associated with low BMD, this suggests that the beneficial effect of denosumab on BMD in patients with inflammatory diseases may be weaker than in controls. Available data are not sufficient to determine whether there is a difference in the therapeutic effect of denosumab on BMD in patients with inflammatory arthritis such as RA compared with a control group of patients without arthritis. In this study, we found that RA patients showed similar percent changes in BMD as assessed by aBMD and T-scores of the lumbar spine compared to controls after denosumab treatment for 2 years. However, the control group showed a slightly increased trend in BMD of the femur neck and total hip compared with RA patients. The present study found an increase in BMD of 5.31% at the lumbar spine and 4.13% at the femur neck after 2 years of denosumab treatment in RA patients, whereas a 9.2% increase in BMD of the lumbar spine and 6.0% increase at the total hip were noted in patients treated with denosumab for 36 months in the FREEDOM trial.^[13]^ A comparison of the absolute values and percent changes in BMD after denosumab treatment between RA patients and subjects participating in the FREEDOM study suggests that the treatment effect of denosumab on BMD in RA patients may be equivalent to the effect in the general population, even considering the differences in treatment duration and presence of chronic inflammation.

Although the pathogenic mechanism and diagnostic criteria of osteoporosis have been well understood, novel emerging and investigational therapeutic agents against osteoporosis-related mediators or signal pathways are under development. Currently available antiresorptive drugs such as bisphosphonates and SERMs are used widely to reduce the risk of vertebral, non-vertebral, and hip fractures in clinical practice.^[10,11]^ However, if there is no improvement in BMD enough to reduce the risk of fracture despite the administration of these drugs, other treatment options with different mechanisms of action should be considered to decrease the risk of fractures. Novel agents for osteoporosis, including RANKL inhibitor, cathepsin K inhibitor, and anti-sclerostin antibody, are emerging for treatment of refractory osteoporosis.^[10,11]^ Denosumab is the first biologic drug used for osteoporosis treatment that blocks RANKL to suppress bone resorption.^[12]^ Denosumab is used as a first-line drug for patients with a high to very high risk of fractures, according to the American Association of Clinical Endocrinology/American College of Endocrinology clinical practice guidelines for the treatment of postmenopausal osteoporosis.^[33]^ However, anti-osteoporosis treatment with denosumab became available in late 2016 in Korea and was permitted only in patients who had no therapeutic response on BMD by DXA even after taking bisphosphonates or SERMs for more than 1 year due to limited insurance coverage for osteoporosis treatment. The FREEDOM trial was conducted after excluding patients who had used bisphosphonates for more than 3 years or had used SERMs within the last 6 weeks to minimize their impact on bone metabolism.^[13]^ The results of the FREEDOM trial are indicative of the effect of denosumab alone without the effect of the preceding treatment drugs. In clinical practice, it may be more common to administer other osteoporosis drugs prior to denosumab. Insufficient research data are available on the effect of denosumab on BMD in patients who received bisphosphonates or SERMs prior to denosumab administration. Kaneko *et al* demonstrated that the efficacy of denosumab on BMD in a switch group after long-term use of bisphosphonates is comparable to that in an osteoporosis-treatment-naive group of RA patients.^[34]^ We found that the use of bisphosphonates or SERMs prior to denosumab treatment did not influence denosumab-related therapeutic effects in RA patients. This suggests that denosumab can be a useful treatment option in patients who do not exhibit a treatment response regardless of previous use of anti-osteoporotic drugs.

There are several limitations in interpreting results presented from our study. First, some bias related to selection of subjects enrolled in this study or cofounding factors cannot be avoided due to the retrospective nature of the study. However, there were no statistical differences in baseline characteristics, including demographic data, laboratory results, and BMD values, with the exception of previous fractures and vitamin D levels. The impact of confounding variables that can affect the analysis to some extent appears to be limited. Second, the treatment effect of denosumab was evaluated only by percent changes in aBMD and T-scores, but available data on bone turnover markers or new-onset fractures were not presented. Bone turnover markers reflect microarchitectural alterations in bone quality and play a complementary role in the assessment of changes in BMD when evaluating the risk of fractures.^[35]^ Clinical studies with a prospective study design are needed to determine the treatment effect of denosumab according to standard treatment practices in real-world clinical settings.

In conclusion, denosumab treatment for 1 year appears to have a lower therapeutic effect at the femur neck and total hip of female RA patients compared with control group. But denosumab treatment for 2 years showed similar therapeutic effect between RA patients and controls at the lumbar spine and femur neck. This suggests that denosumab treatment generally shows equivalent treatment effects on BMD regardless of the presence or absence of RA. In addition, the use of either bisphosphonates or SERMs prior to denosumab treatment did not affect the treatment efficacy on BMD in RA patients with postmenopausal osteoporosis. Based on these findings, denosumab treatment in RA may be a clinically useful option for reducing fracture risks and increasing bone mass with equivalent efficacy in healthy subjects.

## Acknowledgments

We would like to thank clinical research nurses Hyo-Jeong Lee and Eun-Ha Kim in charge of collecting data collection from study population.

## Author contributions

**Conceptualization:** Seong-Kyu Kim, Jung-Yoon Choe.

**Data curation:** Seong-Kyu Kim, Ji-Won Kim, Hwajeong Lee, Sung-Hoon Park, Jung-Yoon Choe.

**Formal analysis:** Seong-Kyu Kim, Jung-Yoon Choe.

**Investigation:** Seong-Kyu Kim, Hwajeong Lee, Sung-Hoon Park, Boyoung Kim.

**Methodology:** Seong-Kyu Kim, Jung-Yoon Choe.

**Software:** Seong-Kyu Kim, Jung-Yoon Choe.

**Supervision:** Seong-Kyu Kim.

**Writing – original draft:** Seong-Kyu Kim.

**Writing – review & editing:** Seong-Kyu Kim.
